# Zoo Studies: Living Collections, Their Animals and Visitors

**DOI:** 10.1017/awf.2023.81

**Published:** 2023-09-11

**Authors:** Ellen Williams

**Affiliations:** Harper Adams University, UK

*Zoo Studies: Living Collections, Their Animals and Visitors* is proclaimed by the author to be an attempt to discuss the breadth of research carried out on zoo animals themselves and the zoological industry more generally, drawing the reader to a host of different topics that are highly relevant within the zoo world. It is written by Dr Paul Rees, who is a zoo scientist previously from the University of Salford. Rees is extensively published in this field and the book refers to much of his previous work with elephants.

As is its aim, the book is multifaceted, covering a range of appropriate topics. Whilst the book cannot possibly cover all of these areas in detail, it gives a good overall introduction to the topics which would be of relevance to those interested in zoos and/or the study of zoo animals. This is no mean feat given the breadth and depth of knowledge now available in this growing sector. Chapters cover a suite of subject matter, including organisation and management of zoos; the animals themselves – caring for animals at an individual level, considering their welfare, enrichment and training of animals; animal management at a broader level including *in situ* and *ex situ* population management and animal health and nutrition; humans in zoos – including who attends zoos and why they attend zoos, visitor behaviour in zoos and their impacts on animals; and finally wider consideration of zoos including ethics, the contribution of zoos to zoology and the future of zoos. Although all of the topics are relevant, the order of delivery jumps about and seemingly doesn’t follow a particularly logical order. Some topics, such as research, and human-animal interactions, crop up in multiple places. The contents page is very detailed though so I would recommend readers utilise that to identify topics of interest and use this as a reference book rather than attempting to read cover-to-cover.

As the author states, the research included ranges from historic to present day, however it would be an almost impossible task to be exhaustive, and so the chapters provide a good starting point for further investigation. That being said, there are some areas where more information would have provided a more thorough review of the topic, and perhaps be more representative of the wealth of work undertaken by modern zoos (either directly or through collaboration with other individuals or establishments). In these areas I would suggest the reader undertakes wider reading of the topic. Some areas of note include the first chapter, ‘zoos and research’ and chapter 3, ‘zoos and education.’ One of the concluding sentences in the first chapter suggests that most zoo research focuses on animals, which I would suggest is an oversimplification of the vast range of research undertaken by and within zoological collections worldwide in the present day. The section on ‘accessibility in zoos’ ideally needed to cover the range of work zoos do for all persons. Accessibility within zoos is a growing topic, and I would urge readers to familiarise themselves with the wealth of work currently being carried out by zoos to support this. The chapter on ethics in zoos would have benefited from the inclusion of information from (or at least reference to) Jenny Gray’s book entitled *Zoo Ethics: The Challenges of Compassionate Conservation* and the chapter on animal training could have been readily supplemented by *Zoo Animal Learning and Training*, edited by Vicky Melfi, Nicole Dorey and Samantha J Ward. There is brief mention of the Five Domains, but no reference to the most recent iteration of the Five Domains model published by Mellor and colleagues in 2020 (available at: https://www.mdpi.com/2076-2615/10/10/1870). As this is so integral to the work of zoos, it is a shame it was not included. Finally, it is prudent to note that whilst the four missions of modern zoos, namely conservation, education, research and recreation were covered within the chapters these were not explicitly labelled as modern zoo missions. Whilst these may change in the future, it is likely they will be added to rather than replaced.

In conclusion, I would recommend this book to those interested in zoos/the work of zoos/animals in zoos, but predominantly as a book that introduces relevant topics/areas for consideration and offers direction for areas to further investigate, rather than as a sole provider of information on the complexities of modern zoos. Whilst literature is cited throughout, there is a growing body of research which can complement this work. Chapter one is a very useful place to support wider exploration of the literature, as Rees concisely summarises the range of different places zoo research is widely published, including naming currently popular zoo journals, and also a number of other journals which frequently publish zoo-themed research.

